# Inflammatory activation of the FcγR and IFNγR pathways co-influences the differentiation and activity of osteoclasts

**DOI:** 10.3389/fimmu.2022.958974

**Published:** 2022-09-06

**Authors:** Bettina Groetsch, Elisabeth Schachtschabel, Philipp Tripal, Benjamin Schmid, Ana-Suncana Smith, Georg Schett, Aline Bozec

**Affiliations:** ^1^ Department of Internal Medicine 3, Friedrich-Alexander-University Erlangen-Nürnberg (FAU) and Universitätsklinikum Erlangen, Erlangen, Germany; ^2^ Optical Imaging Centre Erlangen (OICE), Friedrich-Alexander-Universität (FAU) Erlangen-Nürnberg, Erlangen, Germany; ^3^ Institute for Theoretical Physics, Friedrich Alexander University Erlangen-Nürnberg, Erlangen, Germany

**Keywords:** Osteoclast, FcγR3, IFNγR, rheumatoid arthritis (RA), ITAM signaling, IFNγR signaling

## Abstract

Osteoclasts are polykaryons formed by cell–cell fusion of highly motile progenitors of the myeloid lineage. Osteoclast activity can preserve skeletal strength and bone homeostasis. However, osteoclasts are responsible for bone destruction in rheumatoid arthritis (RA). Fc receptors activated by IgG immune complexes (IC) can boost osteoclast differentiation and bone loss in the course of RA. In contrast, interferon (IFN) γ secreted by immune cells blocks osteoclast activation. Despite their hypothetical importance in the regulation of osteoclast differentiation in RA, the interconnection between the two pathways has not been described so far. Here, we show by total internal reflection fluorescence (TIRF) microscopy that FcγR3 and IFNγ receptor (IFNγR) locate at close vicinity to each other on the human osteoclast surface. Moreover, the average distance increases during the differentiation process. Interestingly, FcγR and IFNγR activation shapes the position of both receptors to each other. Surprisingly, the inhibitory action of IFNγ on *in-vitro* human osteoclast differentiation depends on the osteoclast differentiation stage. Indeed, IFNγR activation in early osteoclast precursors completely inhibits the formation of polynucleated osteoclasts, while in premature osteoclasts, it further enhanced their fusion. In addition, gene expression analyses showed that IFNγR activation on early precursor cells but not on premature osteoclasts could induce FcγR expression, suggesting a co-regulation of both receptors on human osteoclast precursors. Phosphokinase array data of precursor cells demonstrate that the observed divergence of IFNγR signaling is dependent on the mitogen−activated protein kinase (MAPK) downstream signaling pathway. Overall, our data indicate that FcγR and IFNγR signaling pathways co-influence the differentiation and activity of osteoclasts dependent on the differentiation state, which might reflect the different stages in RA.

## 1 Introduction

Bone is continuously remodeled by the bone-forming osteoblasts derived from mesenchymal stem cells and by the bone-resorbing osteoclasts derived from the monocyte lineage. Monocyte fusion into mature multinucleated osteoclasts requires a substantial membrane rearrangement and cytoskeletal remodeling (1). Their activity is essential to preserve normal skeletal strength and bone homeostasis. To do so, osteoclasts attach firmly to the bone surface by forming stable actin rings (1, 2). The whole process of membrane fusion and bone resorption is primarily triggered by RANKL- and inositol triphosphate-induced Ca2+ influx followed by activation of nuclear factor of activated T cells 1 (NFATc1), a master transcription factor for osteoclastogenic gene regulation (3). In detail, osteoclast precursors are stimulated by macrophage colony-stimulating factor (M-CSF) and RANKL. Activation of RANK is the first osteoclastogenic event; it recruits the intracellular tumor necrosis factor receptor-associated factors 6 (TRAF6) to induce downstream cytoskeletal remodeling and functional resorptive activity of osteoclasts (4–6). Functionally, TRAF6 interacts with c-Src kinase to induce nuclear factor kappa B (NF-κB) and mitogen-activated protein kinases (MAPKs), such as c-Jun N-terminal kinase (JNK), extracellular signal-regulated kinase (ERK), and p38 MAPKs, which are involved in osteoclast differentiation (7, 8).

Rheumatoid arthritis (RA) is a chronic autoimmune disease affecting approximately 1% of the world’s population, making it one of the most prevalent autoimmune diseases. It causes the degradation of cartilage and bone tissue. This means chronic pain, severe disability, and increased mortality (9, 10). Inflammatory bone loss in RA is caused by altered bone homeostasis with increased osteoclast generation and activity resulting in accelerated bone resorption (11). Although the reasons for enhanced osteoclast activity have been the focus of extensive research, the triggers that lead to progressive bone loss have not yet been adequately researched. Of note, recently, aside from the systemic effects of inflammatory cytokines, autoantibodies have been described to directly and indirectly influence osteoclasts *via* induction of inflammatory cytokines released by myeloid cells within the synovial joint (12). The most specific autoantibodies associated with RA are antibodies against citrullinated proteins (ACPAs). The presence of ACPA in established RA is strongly linked to bone loss (12, 13). ACPA represents complexes of IgG autoantibodies described to induce FcγR ITAM signaling and thereby osteoclastogenesis (14, 15). Indeed, osteoclast development is strongly dependent on co-stimulatory signals provided by the accessory protein FcRγ chain (used by FcγR) to enhance the effects of RANKL signaling by amplifying calcium influx required for NFATc1 activation. Human osteoclasts and their precursors possess three classical activating FcγR, namely, FcγR1, FcγR3A, and FcγR3B, which differ in their IgG binding capacity. FcγR1 is the only known high-affinity FcγR that is able to bind uncomplexed IgG, while all the other FcγRs need the crosslinking effects of immune complexes to become activated (12, 16). IgG autoantibodies strongly induce FcγR3 clustering on osteoclasts, leading to rapid internalization of FcγR3 and activation of ITAM signaling pathways by ITAM tyrosine phosphorylation through Src-family kinases (17). This consequently leads to the recruitment of Syk or ZAP-70 kinases to stimulate downstream ITAM/PLCγ signaling (3, 18). The cooperation between RANK and ITAM signaling cascades leads to sustained PLCγ2 phosphorylation required for its efficient activation and subsequent Ca2+ oscillations that works as a key inducer of osteoclastogenesis in RA (19).

In addition to the direct action of autoantibodies on osteoclastogenesis, the release of inflammatory cytokines within the RA synovium upon autoantibody stimulation enhances osteoclast differentiation and function. Synovial inflammation is a hallmark of RA; therefore, the understanding of synovial processes and pathophysiology might be the best way to approach the pathogenesis of RA. Interferon (IFN)γ is an important cytokine during immune inflammation, which influences bone resorption. The fact that MHC class II variations are the strongest genetic risk factor for RA raises the hypothesis that the MHC-II-inducing cytokine, IFNγ, contributes to the development of RA (20). However, its role on osteoclast differentiation is controversially discussed. IFNγ activates the classical JAK–STAT1 pathway to initiate the ubiquitin–proteasome leading to the degradation of TRAF6. Due to TRAF6 degradation, the downstream transcription factors, including NF-κB and JNK, are inhibited leading to reduced osteoclast formation (21, 22). Moreover, IFNγ also inhibits the expression of RANK and NFATc1 (21, 22). Despite these observations, the opposite functions of IFNγ have been described depending on the model used (23–26).

Of note, a possible structural and functional cooperation of the IFNγ receptor (IFNγR) and FcRγ/ITAM adapter on dendritic cells (DCs) and macrophages was recently described (27). Therefore, we hypothesized that the inflammatory milieu within the RA synovium co-stimulates osteoclasts by ACPA autoimmune complexes and IFNγ; thus, FcγR-ITAM and IFNγR signaling co-influences the differentiation of monocytes into active bone-resorbing osteoclasts.

## 2 Material and methods

### 2.1 Human peripheral blood mononuclear cell isolation and osteoclast differentiation

Human peripheral blood mononuclear cells (PBMCs) were isolated according to Steffen et al. (28). In brief, 30–40 ml of fresh human blood containing anticoagulant is diluted 1:1 with PBS, and PBMCs will be isolated by a Ficoll gradient. PBMCs form a small ring lying between the Ficoll layer and the upper part, containing a mixture of blood plasma and PBS. The isolated PBMCs were washed in several steps with PBS/EDTA to get rid of the remaining platelets. Finally, the isolated PBMCs could be counted and plated at a density of 3 × 10^6^ PBMCs/ml in adherence medium (α-MEM, 1% penicillin/streptomycin, 1% FBS) in 500, 250, or 100 μl per well for 24-, 48-, and 96-well cell culture plates, respectively. Cells were incubated for 1 to 2 h at 37°C and 5% CO_2_. After this preincubation, the adherence medium is changed into an osteoclast differentiation medium (α-MEM, 1% penicillin/streptomycin, 10% FBS) containing 1 ng/ml of recombinant human TGF-ß, 30 ng/ml of recombinant human M-CSF, and 5 ng/ml of recombinant human soluble RANKL. The osteoclast differentiation medium was changed every 2 days.

### 2.2 TRAP staining and resorption assay

For tartrate-resistant acid phosphatase (TRAP) staining, 0.3 × 10^6^ peripheral blood mononuclear cells (PBMC)/well were cultured in 96-well plates in 100 µl/well of osteoclast differentiation medium containing 1 ng/ml of recombinant human TGF-ß, 30 ng/ml of human M-CSF, and 5 ng/ml of human RANKL at 37°C and 5.5% CO_2_. The medium was changed every 2 days. The cells were stimulated either on day (d)2, d4, and d6 or only on d6 with 2.5 µg of ACPA, 5 ng of recombinant IFNγ, or both. IgG ACPA was isolated from rheumatoid arthritis patient serum by MCV-Sepharose Columns (Orgentec, Mainz, Germany). On d7, the osteoclasts were stained with a tartrate-resistant acid phosphatase kit TRAP (Sigma, Taufkirchen , Germany) according to the manufacturer’s instructions. In short, the cells were washed with PBS following fixation for 3 min in 100 µl of fixation buffer (25 ml of citrate buffer + 65 ml of acetone + 8 ml of 37% PFA). The cells were stained for around 5 min in 100 µl of staining solution. After staining, the osteoclasts were washed and kept in PBS/glycerol 1/1 in the dark until analysis.

For the resorption assay, 0.3 × 10^6^ PBMCs/well were cultured on calcium phosphate (CaP) (0.12 M of Na2HPO4, 0.20 M of CaCl_2_ pH 7.4)-coated 96-well plates in 100 µl/well of osteoclast medium containing 30 ng/ml of human M-CSF and 5 ng/ml of human RANKL at 37°C and 5.5% CO_2_. The cells were treated as mentioned for the TRAP staining. On d7, the osteoclasts were lysed with dH_2_O, and the plates are incubated with 5% sodium hypochlorite (MilliporeSigma, Taufkirchen, Germany) for 5 min. Afterward, the plates were washed and dried for 5 h at room temperature.

All images were acquired with the Keyence fluorescence microscope, and quantification of osteoclast number and percentage of the resorbed area was performed with the ImageJ software.

### 2.3 Immunofluorescence staining in osteoclasts and total internal reflection fluorescence microscopy

For immunofluorescence, 1.5 × 10^6^ PBMCs/well were cultured in 500 µl/well of osteoclast medium with supplements on 13-mm diameter high-precision glass slides (Marienfeld) in 24-well plates at 37°C and 5.5% CO_2_ until fully differentiated. The medium was changed every 2 days. The cells were stimulated as previously mentioned and then fixed and stained with anti-IFNγR (CD119) and anti-FcγR3 (CD16) antibodies overnight at 4°C. Next, the cells were stained with the secondary antibodies anti-mouse AF488 and anti-rabbit AF568 (Invitrogen, Thermo Fisher Scientific Dreieich, Germany) for 1 h at room temperature. DAPI was used as counterstaining, and cells were embedded in Mowiol for microscopy analyses.

The recorded total internal reflection fluorescence (TIRF) images were analyzed using a custom ImageJ/Fiji plugin. Both AF488 and AF568 channels were processed using a difference-of-Gaussian filter, and IFNγ (AF488) and Fcγ receptors (AF568) were detected as local maxima above a threshold in the filtered images. Distances between the two receptor types were then calculated for each detected IFNγ receptor and the closest located Fcγ receptor.

### 2.4 Human phospho-kinase array and human proteome profiler assay

For protein extraction, 1.5 × 10^6^ PBMCs/well were cultured in 500 µl/well of osteoclast differentiation medium in 24-well plates at 37°C and 5.5% CO_2_. The osteoclasts were supplemented either on d2 or d6 of differentiation with 2.5 µg of ACPA, 5 ng of recombinant IFNγ, or a combination of 2.5 µg of ACPA and 5 ng of recombinant IFNγ. The protein was isolated 45 min post-supplementation.

#### 2.4.1 Human phosphokinase array

For the human phosphokinase array, six wells per treatment were pooled and protein lysates were isolated in 400 µl of lysis buffer 6 (R&D systems, Minneapolis, US). Next, the protein concentration of the cell lysates was determined by a BCA protein assay kit (Bio-Rad, Feldkirchen, Germany). The human phosphokinase array was performed according to the manufacturer’s instruction and 100–150 µg of protein was used for the assay. Images were acquired by a Biostep Chemilumineszenz-Imager CELVIN S 420.

#### 2.4.2 Human proteome profiler assay

For the human proteome profiler assay, the osteoclasts were supplemented as described before on d6 of differentiation. Protein cell lysates from six wells/treatment were isolated after 30 min in scioExtract extraction buffer (Sciomics, Neckargemünd, Germany) containing a protease inhibitor cocktail. The isolated protein samples were directly frozen in liquid nitrogen and kept at −80°C until the measurements. Measurements and data analyses of differentially regulated proteins and phosphoproteins were performed by Sciomics.

##### 2.4.2.1 Sample labeling and incubation

The samples were labeled at an adjusted protein concentration for 2 h with scioDye 2 (Sciomics). After 2 h, the reaction was stopped. The excess dye was removed and the buffer exchanged to PBS. All labeled protein samples were stored at −20°C until use.

The 16 samples were analyzed on 16 scioDiscover antibody microarrays (Sciomics) targeting 1,352 different proteins with 1,821 antibodies. Each antibody is represented on the array in four replicates. The arrays were blocked with scioBlock (Sciomics) on a Hybstation 4800 (Tecan, Austria), and afterwards, the samples as well as scioPhosphomix were incubated.

##### 2.4.2.2 Data acquisition and analyses

Slide scanning was conducted using the PowerScanner (Tecan, Austria) with constant instrument laser power and PMT settings. Spot segmentation was performed with GenePix Pro 6.0 (Molecular Devices, Union City, CA, USA). The acquired raw data were analyzed using the linear models for microarray data (LIMMA) package of R Bioconductor after uploading the median signal intensities. For normalization, a cyclic Loess normalization was applied. For analysis of the samples, a one-factorial linear model was fitted *via* least squares regression with LIMMA, resulting in a two-sided t-test or F-test based on moderated statistics. All presented p-values were adjusted for multiple testing by controlling the false discovery rate according to Benjamini and Hochberg. Proteins were defined as differential for log-fold changes (logFC) >0.5 and an adjusted p-value <0.05. Differences in protein abundance or phosphorylation level between different samples or sample groups are presented as logFC calculated for the basis 2. In a study comparing samples versus control, a logFC = 1 means that the sample group had on average a 21 = 2 fold higher signal than the control group. logFC = −1 stands for 2−1 = 1/2 of the signal in the sample as compared to the control group.

### 2.5 ROS measurements

For the ROS analyses, 0.75 × 10^6^ PBMCs/well were cultured in 250 µl/well of osteoclast differentiation medium in 48-well plates at 37°C and 5.5% CO_2_. The osteoclasts were supplemented either on d2 or d6 of differentiation with 2.5 µg of ACPA, 5 ng of recombinant IFNγ, or a combination of 2.5 µg of ACPA and 5 ng of recombinant IFNγ. After 1 h, the cells were stained with dihydrorhodamine 123 (DHR123, a non-fluorescent dye) that is oxidized to fluorescent R123 within cells in the presence of reactive oxygen species, and it localizes in the mitochondria. The osteoclasts were detached from the wells by 0.05 mM of EDTA for 10 min at 37°C and stained for FcγR3 (anti-CD16) and IFNγR (anti-CD119) cell surface expression. The ROS signal in CD119+CD16+ osteoclasts was analyzed using CytoFLEX flow cytometer and CytExpert software.

### 2.6 RNA extraction and quantitative real-time PCR

For RNA extraction, 0.75 × 10^6^ PBMCs/well were cultured in 250 µl/well of osteoclast differentiation medium in 48-well plates at 37°C and 5.5% CO_2_. The osteoclasts were supplemented either on d2 or d6 of differentiation with 2.5 µg of ACPA, 5 ng of recombinant IFNγ, or a combination of 2.5 µg of ACPA and 5 ng of recombinant IFNγ. After 24 h, the cells were harvested in RNA-Solv Reagent, and RNA was extracted according to the manufacturer’s instructions and reversely transcribed into cDNA using oligo d (T) primers. qPCRs were performed using SYBR select Master Mix (Applied Biosystems). The samples were analyzed in duplicate and normalized to the level of hB2M. The primer sequences for real-time analysis are shown in **Table 1**.

**Table 1:** Primer sequences for real-time analyses.

Name	Sequence forward	Sequence reverse
hTRAP	TGAGGACGTATTCTCTGACCG	CACATTGGTCTGTGGGATCTTG
hCathK	AGAAGACCCACAGGAAGCAA	GCCTCAAGGTTATGGATGGA
hNFATc1	GTCCTGTCTGGCCACAAC	GGTCAGTTTTCGCTTCCATC
hOscar	AGATCGCTCCCCTTCTCTTC	TAGCAGCAGCGGTAACTTCC
hFcγR1A/B	ATGCGTGGAAGGATAAGCTG	GATGCTTTCCCATGCCTGAG
hFcγR3A/B	GTTTCAGCTGGCATGCGG	GGGTGGAGAGGTTTGTCTGG
hIFNγR2	CTGCTCGGGAAGAGGCG	ACCTGATGATGAGGGAGCCT
h casp8	GCAAAGGAAGCAAGAACCCA	CCTGGTGTCTGAAGTTCCCT
h MLKL	AGGACCAAGGAAAGAGGAGC	CGAGAGCTCCTTCCAGACAT
h Bcl2	CCTCGCTGCACAAATACTCC	TGGAGAGAATGTTGGCGTCT
hRIPK3	CCAAATCCAGTAACAGGGCG	TCTTTAGGGCCTTCTTGCGA
hp53	TGGCCATCTACAAGCAGTCA	GGTACAGTCAGAGCCAACCT
hB2M	GATGAGTATGCCTGCCGTGTG	CAATCCAAATGCGGCATCT

### 2.7 Statistical analysis

All statistical analyses were performed using GraphPad Prism software (GraphPad software, La Jolla, CA, USA). Statistical significance was calculated by t-test or one-way ANOVA using GraphPad Prism 8 software. Significance is indicated in the p-values.

## 3 Results

### 3.1 The close proximity of FcγR3 and IFNγR on the osteoclast plasma membrane is dependent on their maturation stage

**Figure 1:**
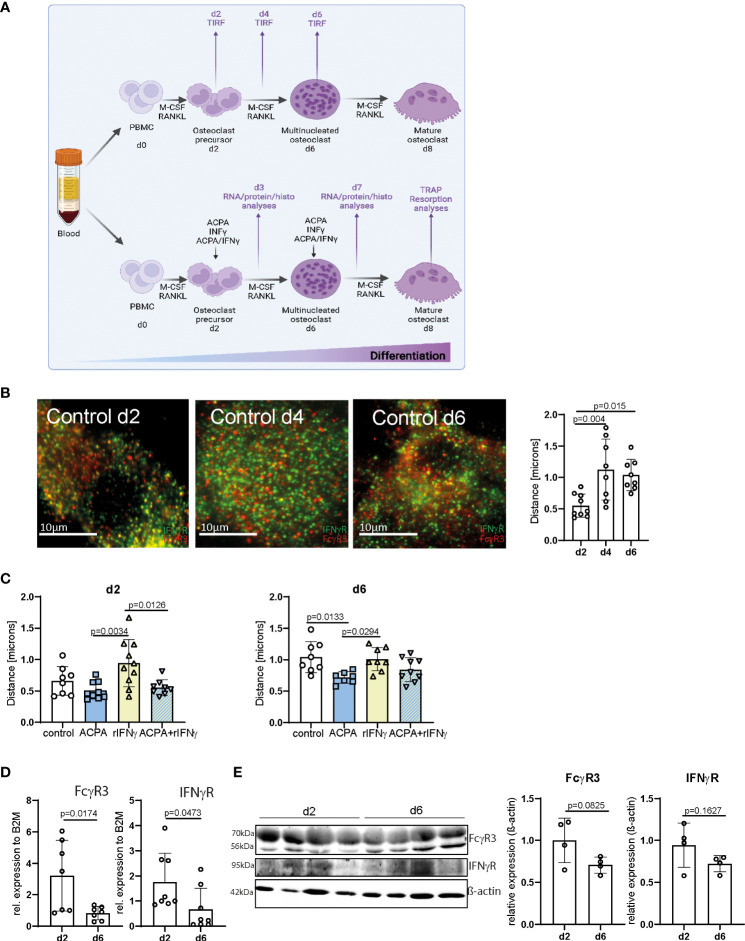
FcγR3 and IFNγR are located in close proximity to each other on the osteoclast plasma membrane. **(A)** Scheme depicting the process of *in-vitro* human peripheral blood mononuclear cell (PBMC) differentiation into mature polynucleated osteoclasts by human M-CSF and human RANKL stimulation for 7/8 days. **(B)** Representative total internal reflection fluorescence (TIRF) microscopy pictures of fixed *in-vitro* differentiated osteoclasts from human PBMCs at the stage of osteoclast precursors day 2 (d2), multinucleated osteoclast (d4), and premature osteoclasts (d6) (left). Distance of FcγR3 (red) and IFNγR (green) measured at the indicated time points by an ImageJ plugin (local maxima) (right) resolution in *x*/*y*/*z* of TIRF images is 200 nm. **(C)** Analyses of receptor distance after stimulation of *in-vitro* differentiated PBMCs on d2, d4, and d6 for 24 h with the indicated stimuli. **(D)** Real-time gene expression analyses in human osteoclasts differentiated *in vitro* for 2 days (d2) or 6 days (d6) with 30 ng/ml of M-CSF and 5 ng/ml of RANKL. Each point indicates the results for a single donor. **(E)** Western blot (left) and quantitative analyses (right) of FcγR3 and IFNγR level in human osteoclasts differentiated *in vitro* for 2 days (d2) or 6 days (d6) with 30 ng/ml of M-CSF and 5 ng/ml of RANKL. Quantitative analyses of Western blot bands were assessed by ImageJ. Data are the mean −/+ s.d. One-way ANOVA **(B, C)** and Student’s *t*-test **(D, E)** were used for the analysis, and the corresponding *p*-values are indicated in the figure.

We and others have demonstrated that Fcγ receptors, and especially FcγR3, activated by IgG immune complexes induce pro-osteoclastic signals (29, 30). The fact that Fcγ− and IFNγ− receptors rely on tyrosine-based phosphorylation involved in cell activation and multimeric endocytic receptor internalization led us to hypothesize that IFNγR and FcγR3 could locate in close proximity during osteoclast differentiation. To test this hypothesis, we co-stained IFNγR and FcγR3 in human PBMCs differentiated into mature polynucleated osteoclasts by human M-CSF and RANKL (**Figure 1A**). By TIRF microscopy, we could show that FcγR3 and IFNγR are closely localized on the human osteoclast surface (**Figure 1B**). Interestingly, the average distance between FcγR3 and IFNγR increases during the differentiation process, especially from d2 to d4 (**Figure 1B**), suggesting a time-dependent function of these receptors upon activation.

Next, we determined the concentration of 2.5 µg of ACPAs and 5 ng of recombinant IFNγ (rIFNγ) with the highest induction potential of osteoclast numbers (**Supplemental Figure 1**), which were then used individually to activate FcγR3 or IFNγR, or the combination of ACPA/rIFNγ at early (d2) or late (d6) steps of osteoclast differentiation for 24 h (**Figure 1A**). Interestingly, FcγR3 and IFNγR activation could individually influence the position of the other receptor (**Figures 1C, D**). While IFNγ stimulation augments the distance between IFNγR and FcγR3, ACPA decreased it. Moreover, ACPA stimulation restores the proximity of the two receptors altered by IFNγ (**Figures 1C, D**). These data suggest that IFNγR and FcγR3 activation can shape their individual distribution.

It is therefore possible that the increasing distance of FcγR3 and IFNγR on multinucleated osteoclasts (d6) might be due to a reduction in either FcγR3 or IFNγR expression on the cell surface. To test this possibility, we analyzed mRNA and protein levels of both receptors on d2 and d6 of osteoclast differentiation (**Figures 1E, F** and **Supplemental Figures 2A–C**). FcγR3 or IFNγR mRNA levels were lower in multinucleated osteoclast than in precursors cells (**Figure 1E**). However, Western blot analyses could only demonstrate a trend of FcγR3 or IFNγR reduction (**Figure 1F** and **Supplemental Figures 2A–C**). Nevertheless, the enhanced distance between FcγR3 and IFNγR might still be explained by a reduction of their surface expression.

### 3.2 The dual function of IFNγ on human osteoclast is dependent on their maturation phase

**Figure 2:**
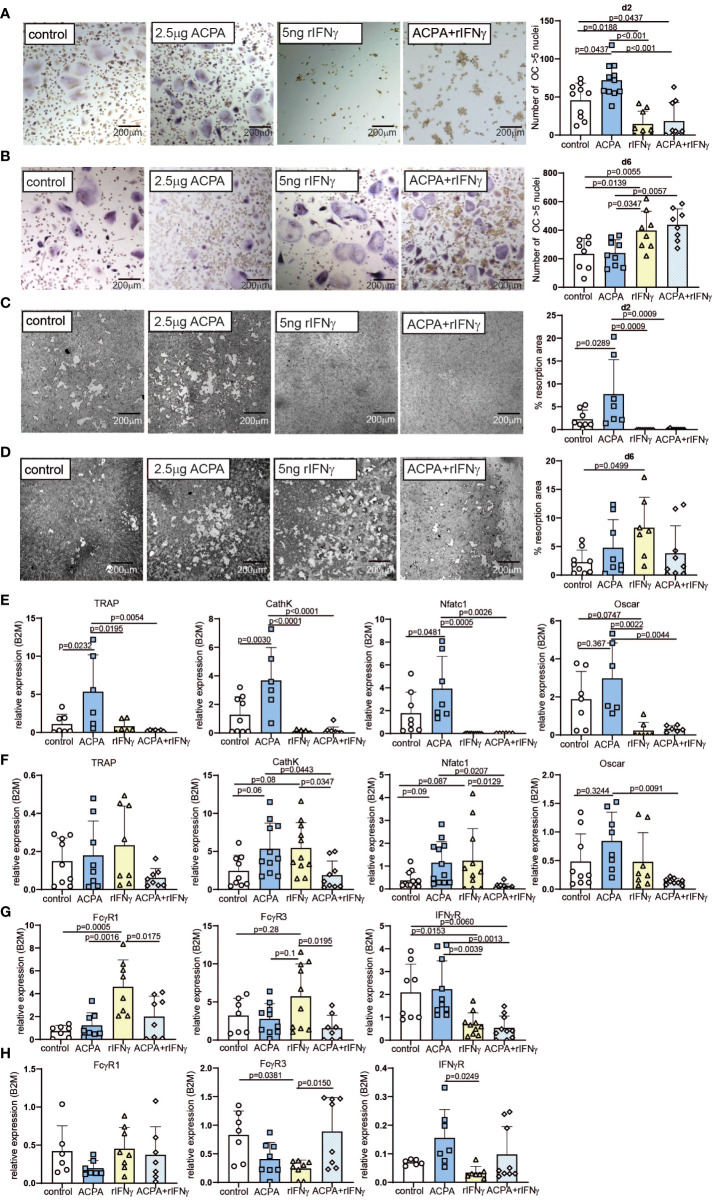
Varying effects of FcγR and IFNγR activation dependent on the stage of osteoclast differentiation. Representative tartrate-resistant acid phosphatase (TRAP) staining (left) and the numbers (right) of *in-vitro* differentiated human peripheral blood mononuclear cells (PBMCs) by M-CSF and RANKL (control) supplemented with 2.5 µg of antibodies against citrullinated proteins (ACPA) or 5 ng of IFNγ at day 2 **(A)** or at day 6 **(B)**. Representative resorption pits (left) and percentage of resorbed area (right) of *in-vitro* differentiated human PBMCs by M-CSF and RANKL (control) supplemented with 2.5 µg of ACPA or 5 ng of IFNγ at day 2 **(C)** or at day 6 **(D)**. Real-time expression analyses of indicated osteoclast marker **(E)** and FcγR1, FcγR3, and IFNγR **(F)** in response to stimulation with 2.5 µg of ACPA or 5 ng of IFNγ at day 2, and real-time expression analyses of indicated osteoclast marker **(G)** and FcγR1, FcγR3, and IFNγR **(H)** in response to stimulation with 2.5 µg of ACPA or 5 ng of IFNγ at day 6 compared to M-CSF- and RANKL-stimulated controls. Data are the mean −/+ s.d. One-way ANOVA was used for the analysis, and the corresponding *p*-values are indicated in the figure.

Based on FcγR3 or IFNγR proximity, we hypothesized that FcγR and IFNγR signaling pathways co-influence the differentiation and activity of osteoclasts. To test this hypothesis, we performed the stimulation of PBMC-derived human osteoclast at early phase d2 and late phase d6 of differentiation with ACPA and/or recombinant IFNγ (**Figure 1A**). We then analyzed osteoclast differentiation by TRAP, osteoclast function by the quantification of resorption and ROS production, and molecular signaling by quantitative PCR. Remarkably, the cell counts of TRAP-positive polynucleated osteoclasts were very low in the d2 treated cells when compared to the d6 treated cells that might indicate increased cell apoptosis in these cultures (**Figures 2A, B**). TUNEL staining for apoptotic cells did not show any major difference in cell apoptosis in d2 and d6 M-CSF- and RANKL-treated cells (**Supplemental Figures 3A, B**). However, there was a significant increase in apoptosis (Bcl2, Casp8) and necroptosis (MLKL, RIPK3) marker expression in M-CSF- and RANKL-stimulated osteoclasts isolated on d6 (**Supplemental Figure 3C**). This increase in apoptotic and necrotic markers in late-stage osteoclasts likely reflects the short life span of mature osteoclasts that undergo apoptosis *in vitro* when fully differentiated.

When stimulated on d2, ACPA supplementation can enhance osteoclast differentiation and function, whereas IFNγ completely blocks osteoclastogenesis, independent of the presence of ACPA in the medium (**Figures 2A, B** and **Supplemental Figures 4A, B**). The analyses of osteoclast marker could confirm that ACPA is a pro-osteoclastic factor, whereas IFNγ fully stops osteoclastogenesis (**Figure 2C**). mRNA quantification of IFNγR demonstrated that IFNγ stimulation leads to a decreased expression of its receptor (**Figure 2D**). Interestingly, IFNγ stimulation can increase FcγR1 and FcγR3 mRNA expression, which is not observed in the co-stimulated cells (**Figure 2D**).

The regulation of FcγR expression by IFNγR stimulation suggests an interconnection in FcγR3 or IFNγR signaling, which we further analyzed at the late phase of osteoclastogenesis. Indeed, similar stimulations were performed on d6 post-differentiation. As already described (8, 9), ACPA supplementation in mature osteoclasts failed to increase their differentiation, resorption, and ROS production potentials (**Figures 2E, F** and **Supplemental Figures 4A–C**). However, to our surprise, IFNγ supplementation induced strong pro-osteoclastogenic differentiation when compared to the control or the ACPA groups (**Figure 2E**). However, the function of osteoclasts as shown by resorption quantification or ROS production was only moderately altered by IFNγ stimulation (**Figure 2F** and **Supplementary Figures 4A–C**). The co-stimulated cells present the same increase of osteoclast differentiation than IFNγ-stimulated cells (**Figures 2E, F**). These observations were confirmed by the analyses of osteoclastic gene expression (**Figure 2G**). Regarding the mRNA levels of FcγRs, IFNγ-stimulated cells on d6 present the opposite effect than IFNγ-stimulated cells on d2, with a reduced FcγR expression after IFNγ stimulation (**Figure 2H**). Since inflammatory cytokines, such as TNF-α, are usually increased in rheumatoid arthritis, we further addressed the effect of rIFNγ on TNF-α-induced osteoclast formation. As expected, supplementation with 20 ng/ml of human TNF-α induced osteoclast differentiation and fusion in pre-osteoclasts (d2) and mature osteoclasts (d6) (**Supplemental Figures 5A–D**). Interestingly, the addition of TNF-α to our osteoclast cultures did not influence the anti- and pro-osteoclastogenic function of rIFNγ in early- and late-stage osteoclast cultures (**Supplemental Figures 5A–D**).

In conclusion, the inhibitory effect of IFNγR signaling or the pro-osteoclastic effect of ACPA is dependent on the osteoclast differentiation stage.

### 3.3 FcγR3 and IFNγR downstream signaling pathways are dependent on osteoclast maturation

**Figure 3:**
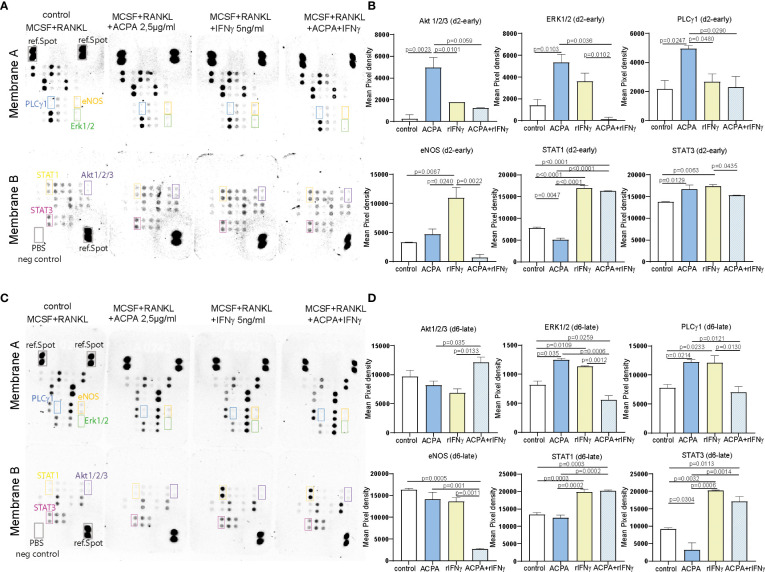
FcγR and IFNγR stimulation influences MAPK signaling dependent on the stage of osteoclast differentiation. Representative pictures **(A)** and calculation of the integrated pixel density of selected spots **(B)** of membranes A and B of the human phosphokinase array developed for 30 min. The membranes were incubated with 150 µg of protein lysates from day 2 osteoclast precursors supplemented with 2.5 µg of antibodies against citrullinated proteins (ACPAs), 5 ng of IFNγ, or a combination of 2.5 µg of ACPA and 5 ng of IFNγ for 1 h compared to non-supplemented controls (M-CSF+RANKL). Representative pictures **(C)** and calculation of the integrated pixel density of selected spots **(D)** of membranes A and B of the human phosphokinase array developed for 30 min. The membranes were incubated with 150 µg of protein lysates from day 6 premature osteoclasts supplemented with 2.5 µg of ACPA, 5 ng of IFNγ, or a combination of 2.5 µg of ACPA and 5 ng of IFNγ for 1 h compared to non-supplemented controls (M-CSF+RANKL). Data are the mean −/+ s.d. One-way ANOVA was used for the analysis, and the corresponding *p*-values are indicated in the figure.

To further define the signaling pathways that might be affected by FcγR3 or IFNγR activation, proteins from precursor or multinucleated osteoclasts stimulated for 1 h with ACPA, IFNγ, or its combination were analyzed by the human phosphokinase array allowing the quantification of 37 kinases. The signal intensity of the detected spots on membranes A and B of the d2 ACPA-supplemented pre-osteoclasts shows a significant increase for phosphorylation of Akt1/2/3 and ERK1/2 MAPK signaling molecules and for PLCγ1 (**Figures 3A, B**). IFNγ supplementation and co-stimulation of pre-osteoclasts, however, did not induce any significant changes in MAPK and PLCγ1 phosphorylation but strongly induced STAT1 and STAT3 phosphorylation (**Figures 3A, B**). Interestingly, only IFNγ treatment but not ACPA or co-stimulation of d2 precursor cells activated eNOS. Our data, therefore, suggest that IFNγR activation on osteoclast precursors on d2 limits intracellular ROS production and its downstream MAPK signaling but induces the phosphorylation of eNOS, STAT1, and STAT3 (**Figures 3A, B**).

When analyzing d6 stimulated samples, ACPA or IFNγ stimulation could both induce ERK and PLCγ1 phosphorylation, whereas no difference was observed in AKT phosphorylation (**Figures 3C, D**). Here, the combined stimulation leads to a reduced PLCγ1 and ERK phosphorylation (**Figures 3C, D**). IFNγ stimulation on d6 still induces STAT1 and STAT3 phosphorylation but not eNOS (**Figures 3C, D**).

The difference between ACPA and IFNγ downstream signaling observed on d2 and d6 nicely reflects the altered functions of these factors on osteoclastogenesis. Taken together, our phosphokinase data further suggest that the osteoclast maturation stage defines IFNγR downstream signaling and thereby the inhibitory effect of IFNγ.

### 3.4 Phosphoproteome analyses of premature osteoclasts show distinct phosphoprotein cluster for IFNγR and FcγR3 activation

To further understand the mechanistic role of how IFNγR activation in the multinucleated cell could induce osteoclastogenesis, we performed an in-depth scioPhospho protein profiling in premature osteoclast supplemented on d6 with 2.5 µg of ACPA, 5 ng of IFNγ, or a combination of ACPA and IFNγ compared to non-supplemented controls. ScioPhospho analysis covers 1,300 highly relevant proteins that are profiled in a single assay and combines the advantages of a robust expression profiling using scioDiscover with information on phosphorylation status.

**Figure 4:**
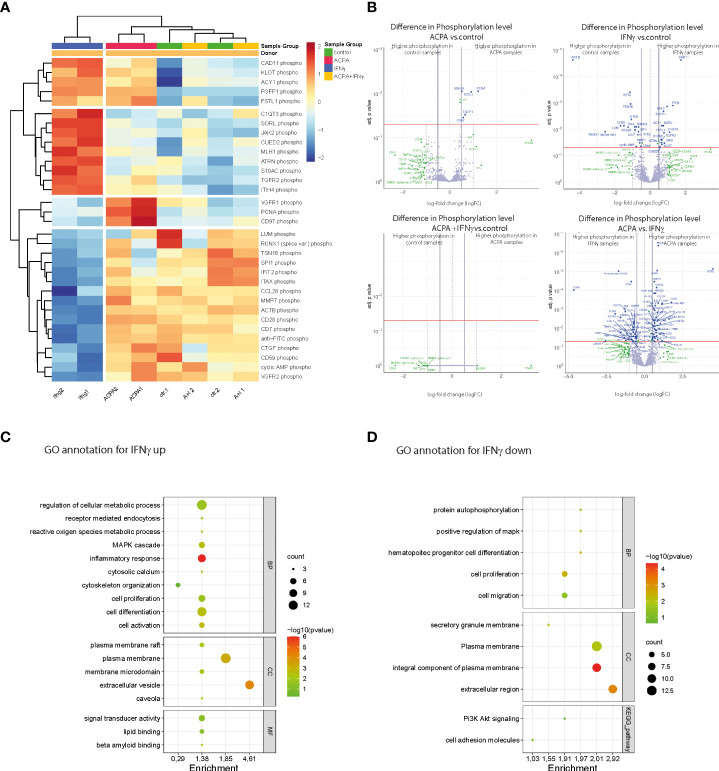
Phosphoproteomic alterations associated with IFNγR and FcγR3 activation at the late stage of osteoclast differentiation. **(A)** Heat map showing the hierarchical cluster analyses of phosphoproteins in human osteoclast cultures supplemented with antibodies against citrullinated proteins (ACPAs), IFNγ, and IFNγ+ACPA at day 6 for 45 min compared to non-supplemented controls. **(B)** Volcano plot showing the differential phosphoprotein expression between the different treatments compared to control and differential phosphoprotein expression between the ACPA treatment and IFNγ treatment. The volcano plot visualizes the *p*-values (adjusted for multiple testing) and corresponding log-fold changes (logFC). A significant level of adjusted *p*-value = 0.05 is indicated as a horizontal red line. The logFC cutoffs are indicated as vertical lines. Proteins with logFC >0.5 and a significant adjusted *p*-value are defined as differential and displayed with blue names. Proteins indicated with green names feature a logFC >1 while not reaching the significance threshold or feature a significant difference while not reaching the logFC threshold. **(C)** Functional annotation clustering (GO term analyses by DAVID Bioinformatics Resources) for proteins with increased phosphorylation and **(D)** for proteins with decreased phosphorylation in response to IFNγ treatment. Graphical bubble plots were designed by the SRplot software. BP, biological process; CC, cellular component; MF, molecular function. All presented *p*-values were adjusted for multiple testing by controlling the false discovery rate according to Benjamini and Hochberg.

Based on the hierarchical cluster analyses of differentially phosphorylated proteins, IFNγ- and ACPA-treated cells have distinct clusters compared to control. Surprisingly, the combination of IFNγ and ACPA treatments shows more similar proteome pattern than control cells (**Figure 4A**), suggesting a negative compensatory mechanism within the two activated signaling pathways. Within the different activation clusters, we could define altered genes essential for osteoclastogenesis such as *ITHI4, Fgfbp1, PCNA, and CCL28* (**Figure 4A**). Volcano plot analyses of the different treatment groups compared to non-treated cells depicted a strong induction of protein phosphorylation in IFNγ-treated cells but only a few in ACPA-treated cells (**Figure 4B**). When comparing the combination of IFNγ and ACPA treatment to the non-treated cells in the volcano plot, we could confirm that both phosphoproteome patterns are similar (**Figure 4B**).

Next, we performed functional gene ontology (GO) annotation clustering of proteins with increased (**Figure 4C**) or reduced (**Figure 4D**) phosphorylation in response to IFNγ treatment compared to the control group. The GO annotation showed an increased phosphoproteome for cellular metabolic (ROS) processes, MAPK signaling, cytoskeleton organization, cell differentiation, and activation that are all in line with the observed stimulatory effect of IFNγ on mature osteoclasts (**Figure 4C**). Interestingly, GO annotation also identified plasma membrane rafts and caveola in the cellular components (CCs) as well as lipid binding within the molecular functions (MFs). The latter point to the role of plasma membrane lipids in osteoclast differentiation induced by IFNγ stimulation. The GO annotation for phosphorylated proteins that were downregulated by IFNγ treatment could be mainly clustered to the biological process (BP) of cell proliferation and migration (**Figure 4D**). In conclusion, the phosphoproteome data show that rIFNγ and ACPA stimulation of multinucleated osteoclasts induced distinct downstream signaling events while co-stimulated mature osteoclasts clustered to the control cells, thereby suggesting that FcγR3 activation could blunt IFNγR-induced effects in mature osteoclasts.

## 4 Discussion

In this study, we highlighted the dual role of ACPA and IFNγ signaling in the process of osteoclast differentiation that might reflect interactions occurring in the RA synovium. The observed alterations are linked to independent cellular processes during osteoclast differentiation, fusion, and maturation. IFNγ treatment mainly blocks the process of cell proliferation and activation through ROS and downstream MAPK signaling important in the early stage of osteoclastogenesis, whereas IFNγ treatment activates genes in cellular matrix organization and plasma membrane remodeling, representing crucial steps in osteoclast maturation and bone resorption. Moreover, our data indicate that co-stimulation of both signaling pathways results in a negative compensatory mechanism within the mature osteoclast. Thus, within the inflamed synovium of RA patients, co-stimulation of osteoclasts by ACPA and IFNγ might work as a negative feedback loop to control the fusion of mature osteoclasts. In conclusion, targeting both pathways in RA patients dependent on the stage of disease might be a new concept in the treatment of inflammatory bone loss within the RA synovial joint.

Inflammation can initiate bone loss *via* the action of cytokines. Apart from inflammatory cytokines, however, ACPAs are also a risk factor in the development of bone erosive disease in patients with RA. Even more, it was recently shown that ACPA binds to the FcγR on osteoclast precursor cells to directly promote their differentiation into bone-resorbing osteoclasts (14). In our study, we could confirm the stimulatory effect of ACPA to osteoclast precursor cell maturation. Noteworthy, ACPA drives the differentiation of osteoclast precursor cells into bone-resorbing osteoclasts but had little effect on multinucleated osteoclasts, suggesting that ACPA is mainly enhancing precursor cell proliferation and differentiation. Mechanistically, we could show that ACPA stimulation enhances the downstream MAPK pathway in precursor osteoclasts through increased phosphorylation of AKT and ERK. Corresponding to the published data (31, 32), ACPA thereby regulates osteoclast differentiation by modulating RANKL-induced phosphatidylinositol 3-kinase/AKT and ERK. Of note, in mature osteoclasts, ACPA continuously activates ERK but fails to activate AKT, an important mediator for osteoclast survival (33). However, this observation could not explain why ACPA stimulation of mature osteoclasts had only a little effect on osteoclast differentiation and fusion.

Synovial inflammation is a hallmark of RA, out of the bulk of cytokines present in the synovium; IFNγ is a prominent one within the micromilieu. Moreover, IFNγ is described to drive inflammatory bone loss in RA patients through activation of RANKL secreting immune cells (23). However, its role in inflammatory bone loss is still heavily discussed since *in-vitro* data show a direct inhibitory role of IFNγ on osteoclast differentiation through blockade of the RANKL signaling pathway (26, 31). Here, we show that the direct inhibitory effect of IFNγ on osteoclast differentiation is dependent on the osteoclast differentiation stage. Hence, only activation of the IFNγR on early pre-osteoclasts blocks osteoclast differentiation and activation, whereas IFNγ stimulation of premature osteoclasts even increased the number and the resorption activity of polynucleated osteoclasts *in vitro*. Of note, similar effects were described for LPS-treated osteoclasts. LPS also limits osteoclast differentiation by reducing the expression of RANK receptor in precursor cells but stimulates osteoclastogenesis in RANKL-pretreated cells *via* TNF-α (34–36). With this, the observed dual role of IFNγ in the process of osteoclast maturation might be a universal mode of action. Mechanistically, we observed that IFNγ treatment in osteoclast precursor cells and in mature osteoclasts strongly induced STAT1 and STAT3 phosphorylation when compared to non-stimulated cells.

Under inflammatory conditions in RA, osteoclasts are heavily influenced by ACPA autoantibodies and IFNγ. However, the mechanism for potential signal integration based on the functional coupling of these two distinct signaling pathways in osteoclasts is not yet described. Interestingly, a possible structural and functional cooperation of the IFNγR and FcRγ/ITAM adapter on DCs and macrophages was published by Bezbradica et al. in 2014 (16). Spatial organization of cell surface proteins plays a key role in the process of transmembrane signaling. Receptor clustering and changes in their cell surface distribution are often determining factors in the final outcome of ligand–receptor interactions (19). Here, we could show by TIRF microscopy that both immune receptors seem to be randomly distributed inside the plasma membrane of pre-osteoclasts and premature osteoclasts. In addition, distance measurements show that FcγR3 and IFNγRs are expressed close to each other but do not co-localize on the osteoclast surface. Most interestingly, our data further suggest that the receptor localization in the osteoclast plasma membrane is not static but changes during the differentiation process and is further influenced by FcγR3 and IFNγR activation. However, to exactly define if the distribution of FcγRs and IFNγRs is uncorrelated and determined by the osteoclast membrane structure and size or if the receptors actively move toward or away from each other, further investigations would require calculating the normalized interaction potential. This could be done by subtracting the probability distribution to find an FcγR protein next to an IFNγR protein in a random system with equivalent density. Of note, our protein and RNA expression analyses of FcγR3 and IFNγR in early pre-osteoclasts and premature osteoclasts highlight an increasing distance of both receptors on premature osteoclasts, which might be caused by reduced receptor expression on the osteoclast. These findings are also in line with the published data showing that FcγR3 is downregulated in response to RANKL stimulation (14). Thus, it would be interesting to model the formation of functional immune domains in the plasma membrane by combining the continuum elasticity theory with stochastic dynamics for protein interactions and statistical approaches for intrinsic activity (20, 21).

IFNγ treatment is described to induce FcγR1 but not FcγR3 expression on the cell surface though the molecular pathway is not yet defined (37, 38). Our data could indeed confirm that IFNγR activation on early precursor cells but not on premature osteoclasts strongly induces FcγR1 expression. Even more, we could also show that IFNγ is able to induce FcγR3 expression on osteoclast precursor cells, suggesting a co-regulation of both receptors especially on human precursor cells. Most interestingly, combined activation of IFNγR and FcγR3 did not induce FcγR expression. In consequence, we hypothesize that FcγR3 activation could blunt IFNγR-induced effects in pre-osteoclasts.

The osteoclast plasma membrane possesses a high level of plasticity especially in the process of cell maturation and fusion. Therefore, IFNγR as well as the FcγR may cluster to distinct specialized areas of the plasma membrane to induce receptor uptake processes, intracellular distributions, and thus, distinct signaling outcomes. It is suggested that distinct membrane localization is involved in the modulation of FcγR and IFNγR function in the process of osteoclast differentiation. Most interestingly, our phosphoproteome data showed that IFNγ treatment on premature osteoclasts targets protein phosphorylation important in cell plasma membrane, cellular matrix, and cytoskeleton organization. These cellular processes are all described to be important in late-stage osteoclast maturation and functional bone resorption (2, 39). Moreover, we could make out a cluster of proteins important in lipid metabolic process, indicating that lipid accumulation and distribution within the plasma cell membrane might affect osteoclast growth in the late maturation process that needs to be further addressed. It is becoming clear that in order to understand how signaling pathways interact, one needs to understand the molecular diversity of the signaling components that comprise the pathways and the functional consequences of this diversity (40). Thus, the molecular identity of components that are present in a certain pathway will determine how that pathway will interact with other pathways.

## Data availability statement

The original contributions presented in the study are included in the article/**Supplementary Material**. Further inquiries can be directed to the corresponding author.

## Ethics statement

This study was reviewed and approved by the Ethics committee from the FAU (Friedrich Alexander Universität) Erlangen (Antrag Nr.334_18B). The patients/participants provided their written informed consent to participate in this study.

## Author contributions

BG and AB designed the study and wrote the manuscript. BG and ES performed the *in-vitro* osteoclast differentiation and the following molecular analyses. PT and BS assisted in the experimental setup and performed the TIRF pictures and distance measurements. ASS contributed to the receptor distance measurements and probability calculations. GS contributed to the discussion and manuscript preparation. GS and AB supervised the study and edited the manuscript. All authors contributed to the article and approved the submitted version.

## Funding

This work was supported by a grant from the Dr. Robert Pfleger Foundation; the German Research Foundation BO-3811/5–1, BO-3811/6–1, and FOR2886 TP02; the SPP μBone; the Interdisciplinary Center for Clinical Research grants A77 and J76; the European Research Council Synergy Grant 4D Nanoscope; and the ERC consolidator grant.

## Acknowledgments

We thank Sciomics and especially Camille Lowy and Christoph Schröder for performing the phosphoproteome analyses and their support in the phosphoproteome data analyses and presentation. We thank the Optical Imaging Center Erlangen (OICE) for providing the tools and for assisting us with the TIRF imaging. We also want to thank Christine Zech, Jule Lindörfer, and Franceska Jelas for their technical assistance.

## Conflict of interest

The authors declare that the research was conducted in the absence of any commercial or financial relationships that could be construed as a potential conflict of interest.

## Publisher’s note

All claims expressed in this article are solely those of the authors and do not necessarily represent those of their affiliated organizations, or those of the publisher, the editors and the reviewers. Any product that may be evaluated in this article, or claim that may be made by its manufacturer, is not guaranteed or endorsed by the publisher.
