# Supplementary figures and images for: Inflammatory activation of the FcγR and IFNγR pathways co-influences the differentiation and activity of osteoclasts
Source: Front Immunol. 2022 Sep 6;13:958974. doi: 10.3389/fimmu.2022.958974 (PMC9486546; doi:10.3389/fimmu.2022.958974)

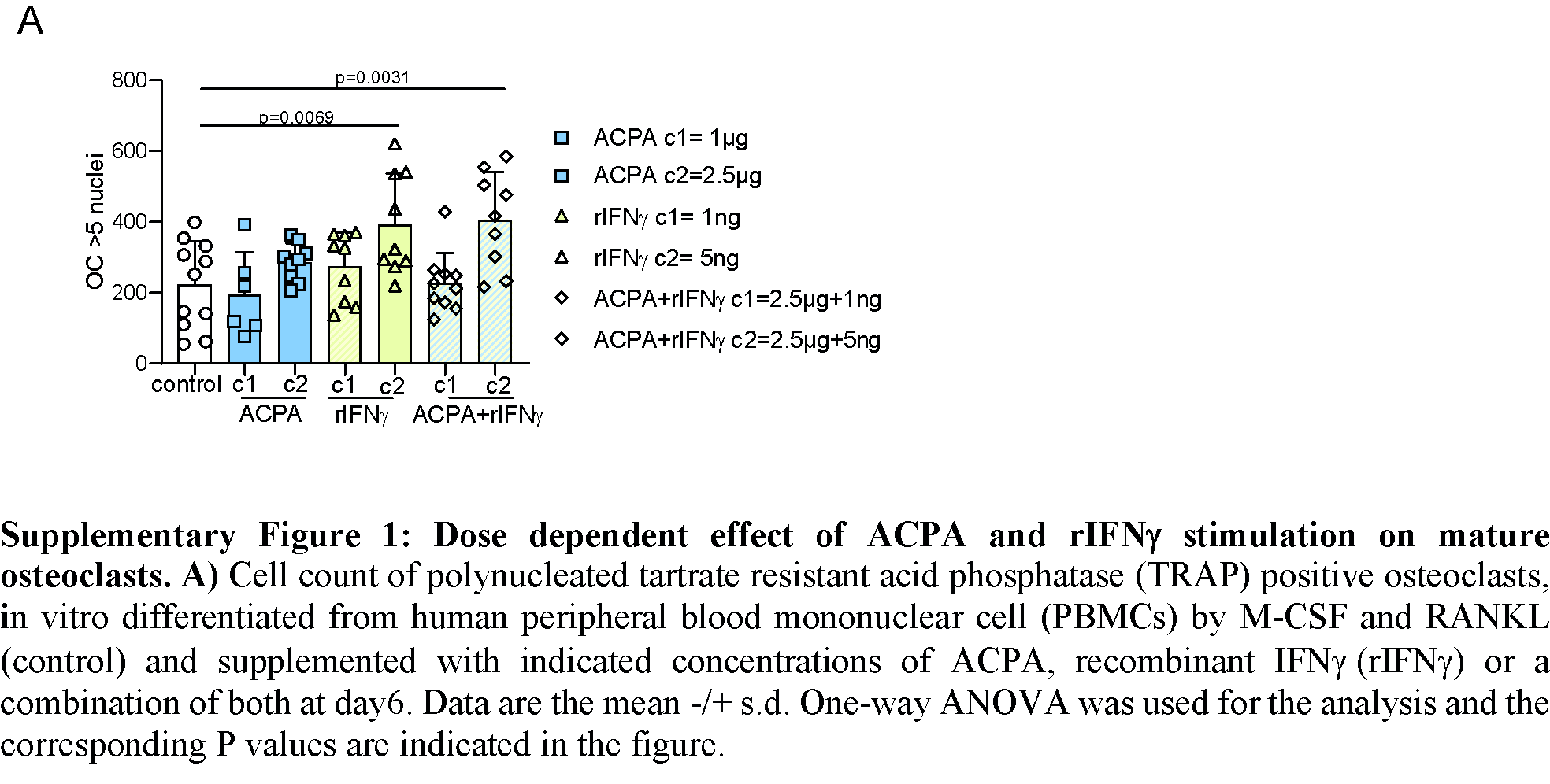

Supplement: Supplementary file 1 [file Image_1.tif]

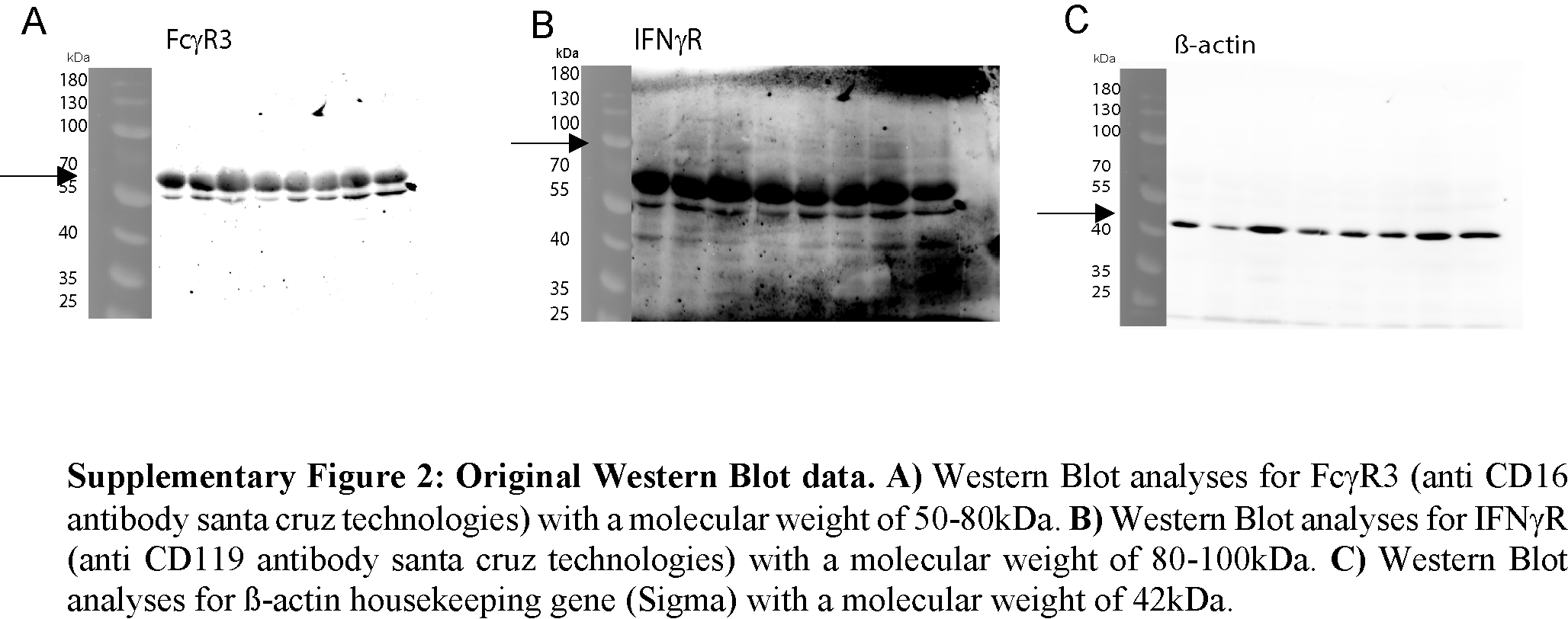

Supplement: Supplementary file 2 [file Image_2.tif]

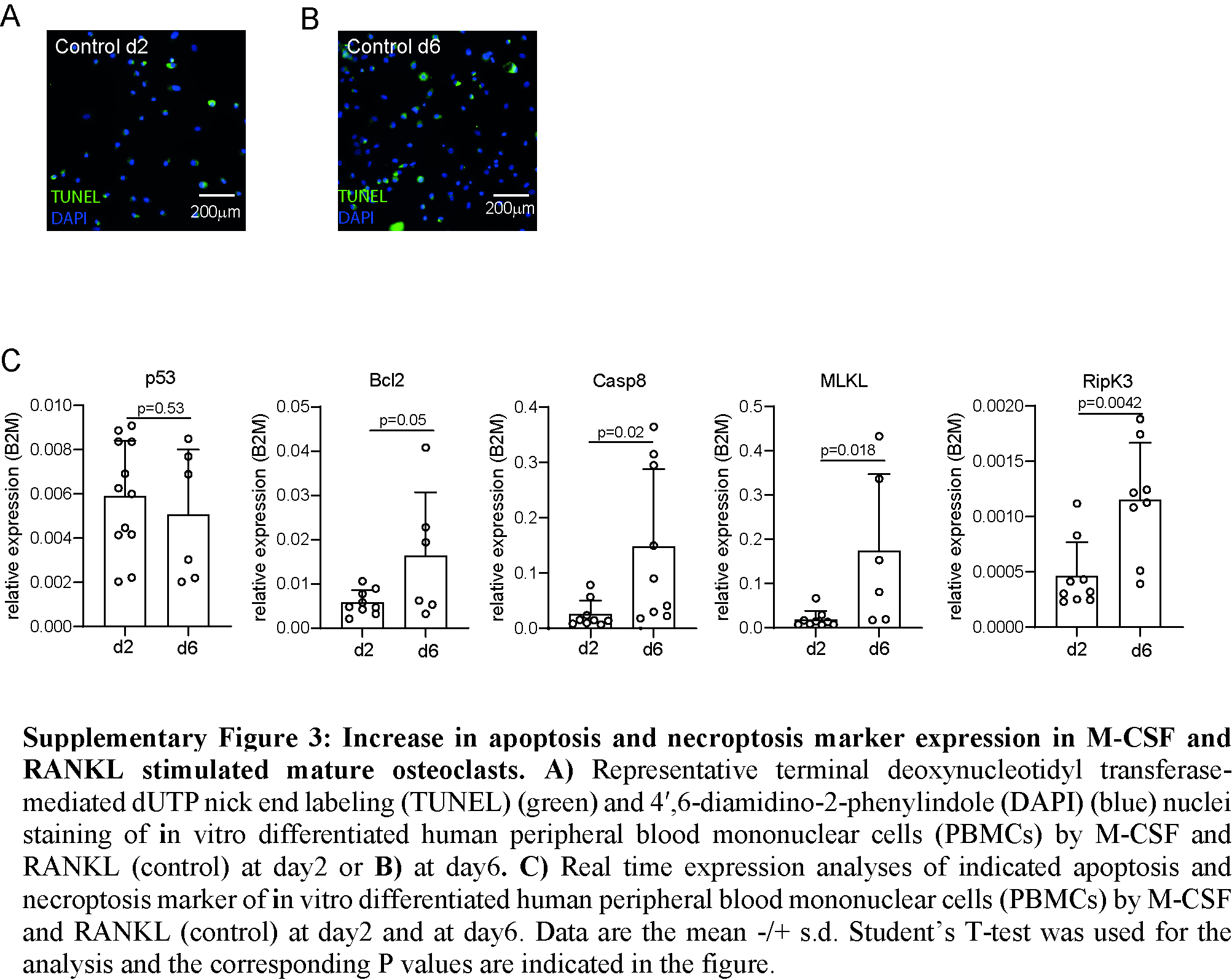

Supplement: Supplementary file 3 [file Image_3.tif]

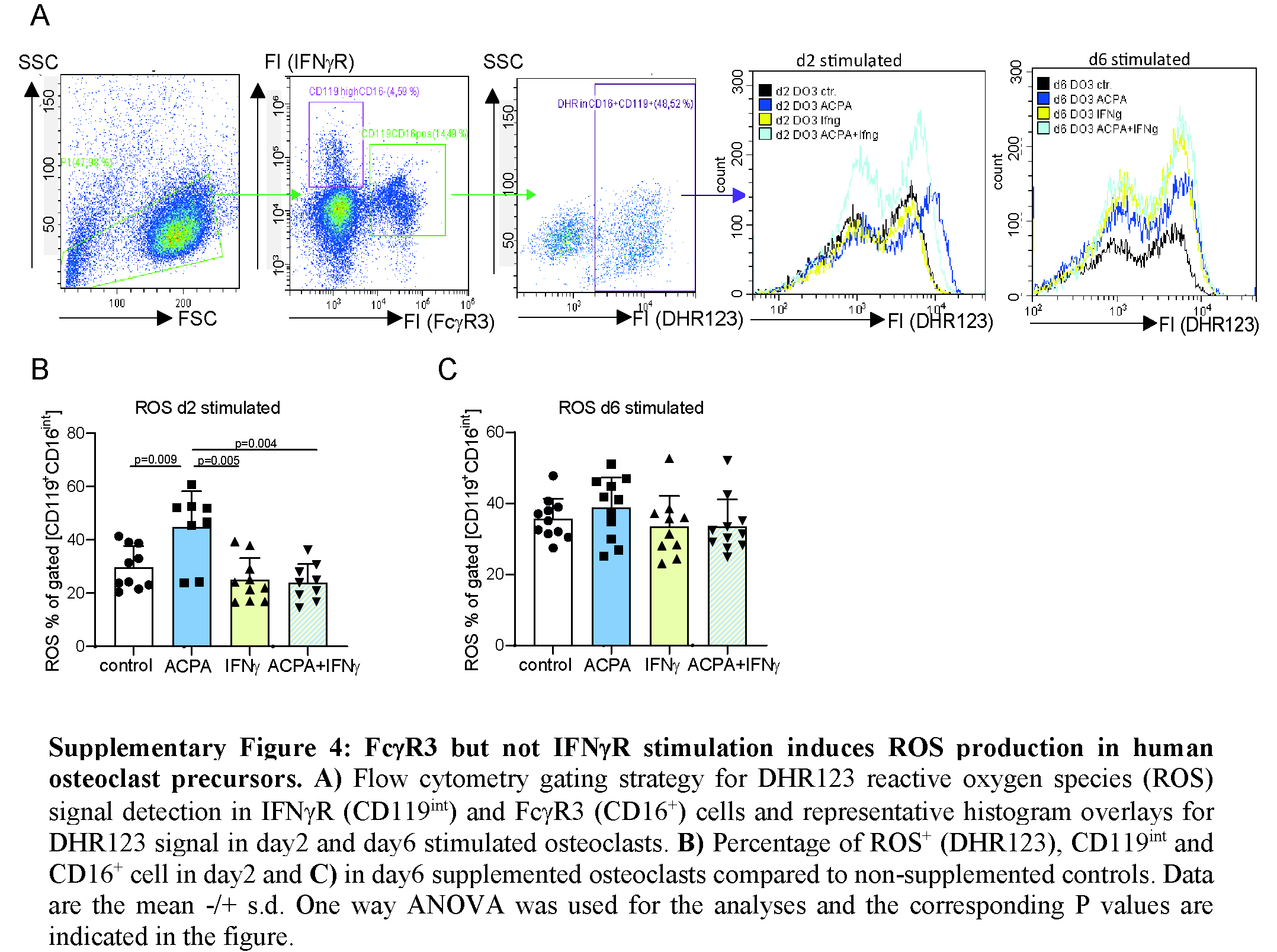

Supplement: Supplementary file 4 [file Image_4.tif]

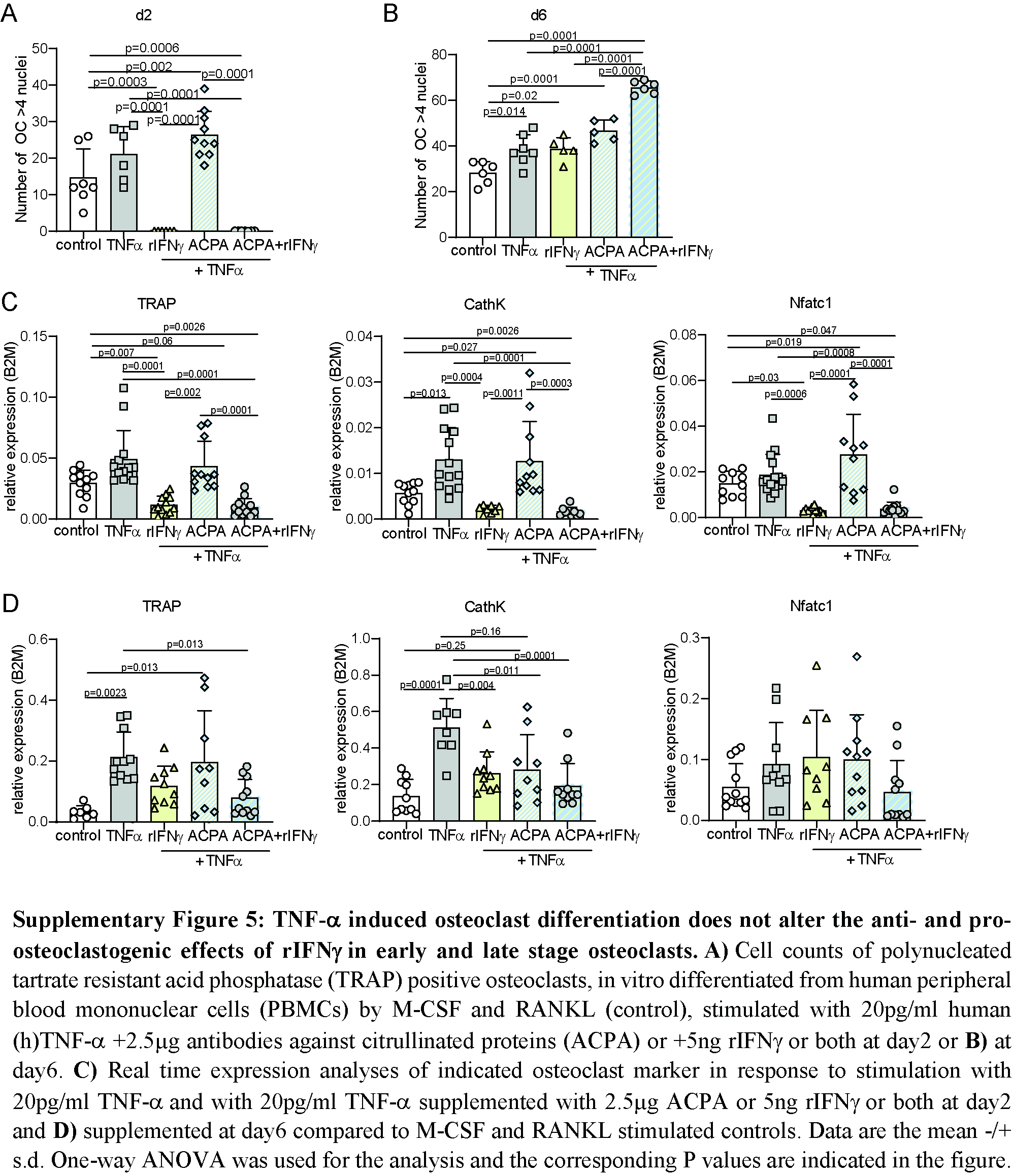

Supplement: Supplementary file 5 [file Image_5.tif]
